# A rare presentation of retroperitoneal liposarcoma presented with jejunal intussusception: An interesting radiological findings

**DOI:** 10.1016/j.radcr.2024.04.021

**Published:** 2024-05-18

**Authors:** Farehah Johari, Andee Dzulkarnaen Zakaria, Rosnelifaizur Ramely, Mohamed Arif Hameed Sultan, Muhamad Hud Muhamad Zin, Shahrunizam Awang Setia, Firdaus Hayati

**Affiliations:** a Department of Surgery, School of Medical Sciences, Health Campus, Universiti Sains Malaysia, Kubang Kerian, Kelantan, Malaysia; b Department of Surgery, Hospital Universiti Sains Malaysia, Kubang Kerian, Kelantan, Malaysia; c Department of Surgery, Faculty of Medicine and Health Sciences, Universiti Malaysia Sabah, Kota Kinabalu, Sabah, Malaysia; d Department of Radiology, Faculty of Medicine and Health Sciences, Universiti Malaysia Sabah, Kota Kinabalu, Sabah, Malaysia

**Keywords:** Atypical lipoma, Jejunal disease, Intussusception, Retroperitoneal liposarcoma, Retroperitoneal neoplasms

## Abstract

A jejunal intussusception as a presentation of retroperitoneal liposarcoma (RLPS) is a rare occurrence. The majority of RLPS are presented as an abdominal mass, however, having a jejunal obstruction is an interesting case. The aim is to describe the management of jejunal intussusception secondary to atypical lipomatous tumours with concurrent RLPS. A 61-year-old lady presented with a sudden onset of intestinal obstruction with 1 month of constitutional symptoms and an enlarging right lumbar mass. Computed tomography showed a small bowel intussusception with diffuse peritoneal and retroperitoneal lipomatosis. Emergency exploratory laparotomy, segmental bowel resection, and partial excision of intraperitoneal mesenteric lipoma were performed. A stage En-bloc resection of the RLPS and right nephrectomy was done later. However, she refused for subsequent surgery. A complete resection is the gold standard in managing RLPS. In this report, the management is rendered not to the standard as the patient first presented with intestinal obstruction requiring emergency reduction with a piecemeal resection. A stage surgery was required to determine a promising prognosis, but the patient refused such surgery. A small bowel intussusception in adults is rare but is mostly caused by a tumor or neoplasm. Early recognition of the complexity of the case should be preempted and referred to the tertiary team for further definitive surgery. Patient exhaustion from the subsequent surgery might hamper the only management available for the case.

## Introduction

Acute intestinal obstruction is a major morbidity and possible mortality especially among the elderly. The aetiology is varied including intussusception. In general, adult small bowel intussusception usually can happen due to benign cause, meanwhile adult large bowel intussusception occurs due to malignant pathology. Adult small bowel intussusception is only represented in 1% of the cases [1]. It is rare to have a tumor in the small bowel to cause adult intussusception [2,3]. The treatment strategy will be challenging especially when dealing with tumors of multiple primaries at the same settings. Herein, we highlight a 61-year-old lady with a sudden onset of intestinal obstruction secondary to jejunal intussusception secondary atypical lipomatous tumor with concurrent retroperitoneal liposarcoma (RLPS) and we describe the management plan for this rare concurrent entity.

## Case report

**Fig. 1:**
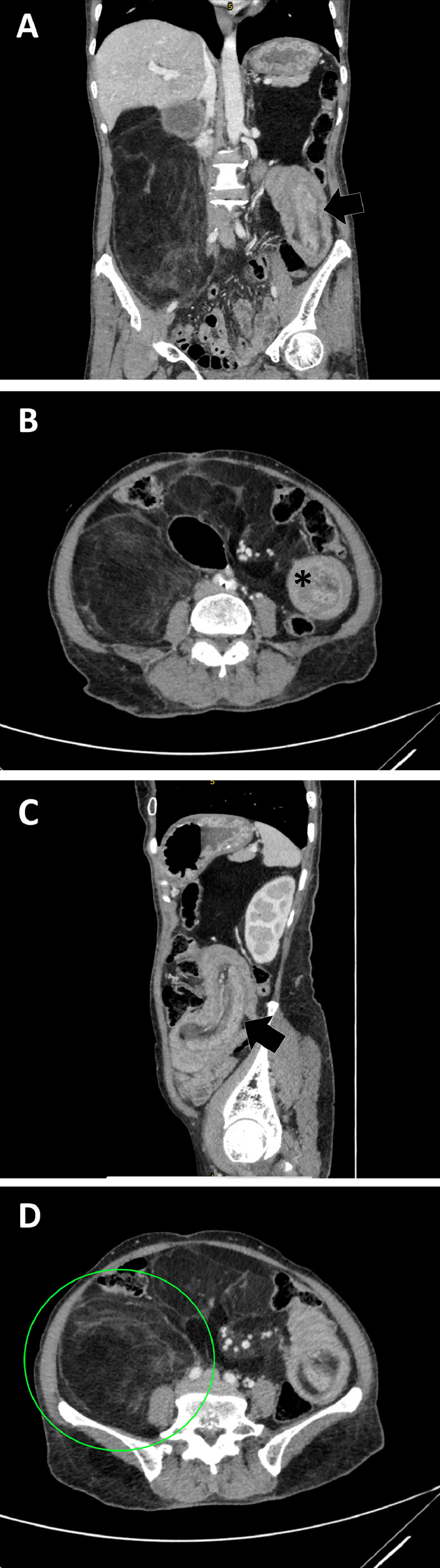
CT abdomen with coronal (A) and axial (B) view showing small bowel intussusception with sausage-like appearance (black arrow) and target sign (asterisk) where fat and mesenteric of small bowel seen within the lumen. It also shows a large right retroperitoneal mass encasing part of the posterior surface of the right kidney. CT abdomen with sagittal (C) view showing a more obvious small bowel intussusception with fat and mesenteric content (black arrow). CT abdomen with axial (D) view showing a different level of cut at L2 level showing the right retroperitoneal mass (green circle) encasing the whole right kidney. It also shows multiple intraperitoneal liposarcoma with similar CT features.

A 61-year-old lady with underlying poorly controlled diabetes mellitus, hypertension, and hyperlipidemia presented at the district hospital with a sudden onset of intestinal obstruction with 1 month of constitutional symptoms and 3 months of gradual increase in the size of the right lumbar mass. Contrast-enhanced computed tomography (CT) of the abdomen and pelvis showed small bowel intussusception (Fig. 1) due to mesenteric lipomatosis with diffuse peritoneal and retroperitoneal lipomatosis.

**Fig. 2:**
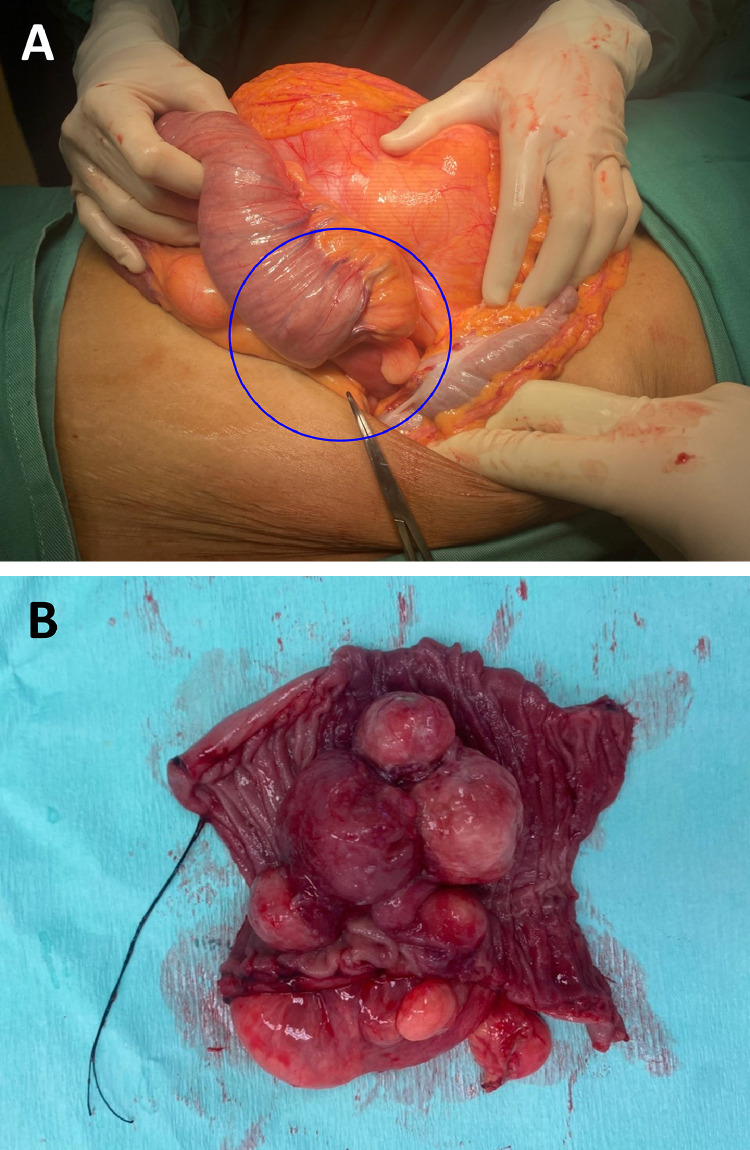
An artery forceps showing a lead point of jejunal intussusception (blue circle) with a large peritoneal lipomatous tumor (A). (B) A cut section of intraluminal lipomatous-like mass of the lead point.

Emergency exploratory laparotomy, segmental bowel resection with primary anastomosis, and partial excision of intraperitoneal mesenteric lipoma were performed on this patient. Intraoperatively noted intussusception at proximal jejunum 20cm from duodenal-jejunal flexure where the lead point was at 25 cm from duodenal-jejunal flexure where resection and anastomosis were done (Fig. 2). There were also multiple intraperitoneal mesenteric lipomas 15 × 15cm where only a partial part of the mass could be excised given no local expertise available. Histopathological examination revealed small bowel intussusception secondary to an atypical lipomatous tumor where the piecemeal resection showed well-differentiated liposarcoma where immunohistochemistry was positive for S100 but negative for desmin, SMA and CD34. This case was referred to our tertiary center where we planned for stage surgical resection.

**Fig. 3:**
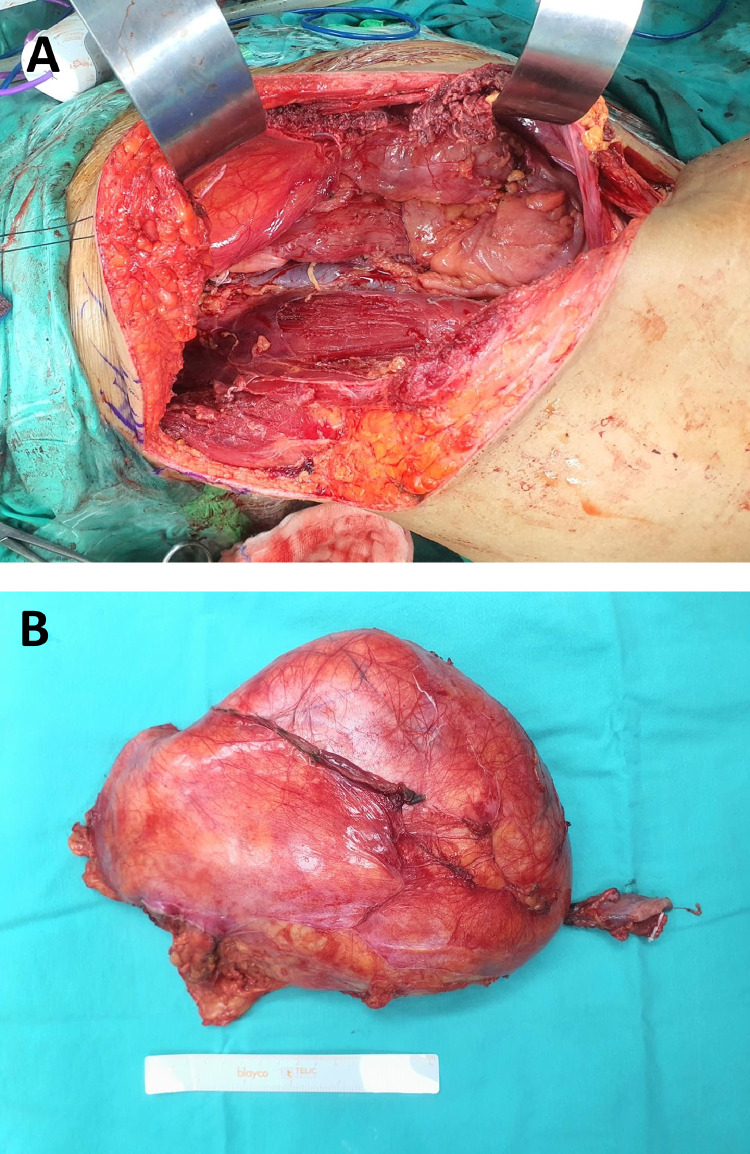
(A) Intraoperative image showing an ilioinguinal approach for retroperitoneal liposarcoma as an easier access to the tumor. (B) A resected retroperitoneal mass encases the right kidney.

After a multidisciplinary meeting decision, we first performed an initial stage en-bloc resection of the RLPS and right nephrectomy (Fig. 3) to avoid prolonged surgery as the patient has multiple comorbid if we were to do complete resection under the same setting. Histopathological examination showed similar findings as previous piecemeal resection which is well-differentiated liposarcoma. We were planning for the completion of intraperitoneal resection soon, however, the patient was not keen on further intervention.

## Discussion

Atypical lipomatous tumors and well-differentiated liposarcoma have similar morphological and genomics and mostly occur in the retroperitoneum or lower trunk [4,5]. There are not many cases reported on the jejunal intussusception secondary to an atypical lipomatous tumor except for only 2-3% of cases [6]. Most of the cases described have no concurrent multifocal retroperitoneal and intraperitoneal well-differentiated liposarcoma even though both are classified as intermediate adipocytic tumors. This made the management of this case challenging for a non-tertiary hospital with no local expertise or consultation available as in our case.

The CT scan is the cornerstone for diagnosis of RLPS. It provides an accurate image of the size, position, and relationship of the tumor to the surrounding organs. Organ displacement or invasion can also be found, as it did in ours for instance [7,8,9]. Despite the mass's location and origin, the CT scan can provide information on tissue composition (such as lipomatous components, calcifications or myxoid elements, and internal necrosis), all of which are crucial for determining the potential RLPS type and other diagnostic options to take into account [10]. When neurovascular and muscle invasion is suspected, magnetic resonance imaging may be helpful. However, it lacks large-scale comparisons which makes it debatable [11]. A biopsy is not always advised, even though it is the gold standard for diagnosis. A percutaneous core needle biopsy is advised only for patients with medically difficult tumors, hematogenous spread, and those being evaluated for preoperative radiotherapy/chemotherapy; it should not be postponed owing to the possibility of tumor seedling.

The gold standard for diagnosing small intestinal lipomas is believed to be an abdominal CT scan. In intussusception, the features of a target and sausage-like appearance will be pathognomonic [12]. The lead point that is composed of a lipoma will exhibit a lipid attenuation (-100 to -50 HU) with avascular hypodense fat density mass. A correct perspective diagnosis will be established by using two-dimensional (2D) CT enterography, which clearly and directly displays the location and range of the tumors as well as the accompanying intussusception.

The dilemma in managing this patient depends on the local setting and expertise. The decision for emergency surgery with piecemeal resection versus initial conservative management with emergency transfer for complete resection at the tertiary centre should be pondered by all managing physicians who are seeing this case. According to the latest guideline by the JNCCN, for resectable disease of atypical lipomatous tumor and well-differentiated RPLS, the first line of management is for resection with an oncologically appropriate margin. In this case, the retroperitoneal resection has R0 resection even if the margin is <1 mm. This is due to the proximity to the nearby organ that may significantly impact the functionality. For R0 disease, if achievable in this case, this management shall follow physical examination with imaging every 3 to 6 months for 2 to 3 years, then every 6 months for the next 2 years, then annually [13]. Systemic therapy is not suggested in a low-grade or well-differentiated tumor [13].

## Conclusion

A small bowel intussusception in adults is rare but is mostly caused by a tumor or neoplasm. Early recognition of the complexity of the case should be preempted and referred to the tertiary team for further definitive surgery. Patient exhaustion for the third surgery might hamper the only management available for the case.

## Data availability

The data used to support the findings of this study are available from the corresponding author upon request.

## Patient consent

The patient's permission was obtained to publish this case report.
